# Viral dsRNA triggers human fetal membrane miR-146a-3p to be packaged into small extracellular vesicles which in turn drives inflammation through activation of Toll-like Receptor 7 and 8

**DOI:** 10.1371/journal.pone.0350139

**Published:** 2026-05-26

**Authors:** Hanah M. Georges, Abigail C. Fischer, Paloma Casanova, Vikki M. Abrahams

**Affiliations:** Department of Obstetrics, Gynecology and Reproductive Sciences, Yale School of Medicine, New Haven, Connecticut, United States of America; Huaqiao University - Quanzhou Campus: Huaqiao University, CHINA

## Abstract

Maternal infection and chorioamnionitis are one of the leading causes of preterm birth and neonatal morbidity. The relationship and mechanisms linking bacterial infections and preterm labor are well researched, however, less is known about the mechanisms involved in how viral infections contribute to preterm labor. Previous work from our group demonstrated that following bacterial triggers, fetal membranes (FMs) express elevated miR-146a-3p which in turn acts as an intermediate danger signal by activating TLR8 to induce a robust inflammatory response. Using an established FM explant model system, the role of this and other TLR7/8-activating miRs in the propagation of viral-induced inflammation was investigated. Following exposure to the viral dsRNA mimic and TLR3 agonist, Poly(I:C), expression of FM tissue TLR7/8-activating miRs (miR-146a-3p, miR-21a, miR-29a, and Let7b) were not elevated. Despite this, in response to Poly(I:C), elevated FM secretion of pro-inflammatory IL-6 and IL-8, and IL-1β was TLR7- and TLR8-dependent. To investigate alternative methods of miR delivery, small extracellular vesicles (sEVs) from FM supernatants were isolated and found to contain elevated levels of miR-146a-3p and miR-21a under Poly(I:C) conditions. Furthermore, Poly(I:C)-induced IL-6 and IL-8 responses were reduced in the presence of an inhibitor of sEV biogenesis/release, and IL-6 and IL-1β production was reduced in the presence of a miR-146a-3p inhibitor. Together, these data suggests that sEVs produced from virally-stimulated human FMs contain and deliver elevated miR-146a-3p which acts as a danger signal to drive perpetuate inflammation via TLR7 and TLR8 activation. This work demonstrates a novel and important role for sEV packaged TLR7/8 activating-miR-146a-3p in FM inflammatory responses to viral infections.

## Introduction

Pregnant women are particularly vulnerable to both bacterial and viral infections. However, maternal infection often goes undiagnosed and untreated, making it one of the largest contributors to maternal morbidity, preterm birth, as well as neonatal morbidity and mortality [1,2]. The contribution of bacterial infections to chorioamnionitis – inflammation of the fetal membranes (FMs) – and preterm birth, are well established [3]. Most mechanistic knowledge about the effects of viral infections on pregnancy and preterm birth comes from studies on *Toxoplasm gondii*, other, *rubella virus*, *cytomegalovirus* and *herpes virus* (TORCH) pathogens; infections which cross the placenta and infect the fetus [4]. However, TORCH pathogens represent a minor fraction of viral infections. With the more recent influenza H1N1 and SARS-CoV-2 pandemics, increased research has shown that viral infections and maternal immune activation can have negative impacts on pregnancy outcomes and on the developing fetus in the absence of vertical transmission [5,6]. A systemic viral maternal infection and maternal immune activation can contribute to cytopathic placental inflammation, placental/trophoblast dysfunction, preterm birth, and developmental disorders [1,2,7–9]. Despite these connections, the mechanisms by which a maternal viral infection may lead to preterm birth, and the mechanism by which fetal membranes respond to viral triggers, are still largely unknown and remains a clinical concern.

The maternal-fetal interface has robust innate immune strategies in place to help protect against invading pathogens. For example, the fetal membranes (FMs) possess the ability to respond to pathogens through pathogen recognition receptors (PRRs) [10–12], leading to an inflammatory response. Once activated, FM expressed PRRs, such as Toll like receptors (TLRs), can trigger inflammation through the production of pro-inflammatory cytokines and chemokines, which can then initiate the production of mediators of membrane weakening and the induction of labor [12,13]. In certain pregnancy pathologies, uncontrolled or untimely inflammation uses the same pathways as normal term labor, resulting in preterm birth [14–16].

Using a model of bacterial infection, our group recently reported a role for endogenous danger signals or DAMPs (damage associated molecular patterns) as intermediate mediators of FM inflammation downstream of an initial TLR sensor [13,17,18]. In previous work, we found that FMs treated with the bacterial components, TLR2-activating peptidoglycan (PDG) and TLR4-activating lipopolysaccharide (LPS), expressed elevated levels of a TLR7/8-activating microRNA [19], miR-146a-3p [20,21]. This increase in miR-146a-3p drives FM inflammation and the production of mediators of membrane weakening in response to bacterial components through activation of TLR8 [13,17]. The identification of such intermediate mediators of inflammation provides a new fundamental understanding of the underlying mechanisms of chorioamnionitis and preterm birth.

While previous bacterial models focused on locally tissue produced TLR7/8-activating miRs as mediators of inflammation, there is potential for alternate delivery methods of pro-inflammatory DAMPs. One potential source of intermediate inflammatory signals are small extracellular vesicles (sEVs) which provide cells the ability to communicate in both autocrine and paracrine manners, and are now known to be critical conduits of cellular communication [22]. Indeed, TLR7/8-activating miRs can be released via sEVs and delivered to target cells to exert a stimulatory effect [19,20,23].

Using an established explant model [13,17], we investigated the role of TLR7/8-activating miRs in driving human FM inflammation in response to viral double stranded RNA (dsRNA). This work reveals an important role for FM-derived sEVs in the delivery of TLR7/8-activating miRs and subsequent inflammatory signaling in response to a viral trigger.

## Materials and methods

### Patient samples

Deidentified human tissue collections were approved by Yale University’s Human Research Protection Program (IRB# 0607001625). Patients undergoing uncomplicated term (38−41 weeks) elective cesarean sections without labor or known infection were consented in writing and FMs were collected by the Yale University Reproductive Sciences (YURS) Biobank approved by Yale University’s Human Research Protection Program (IRB# 1309012696) immediately after delivery [07/01/23–05/01/25]. Minors were not included in this study. FMs were biopsied and cultured as previously described [13,17]. Briefly, FMs were rinsed, biopsied with a 6 mm punch, and placed into 0.4μm cell culture inserts in a 24 well plate (BD Falcon, Franklin Lakes, NJ) containing 1mL of Dulbecco modified eagle medium (DMEM; Gibco, Grand Island, NY) supplemented with 10% fetal bovine serum (Hyclone, Logan, UT) and 1% penicillin streptomycin (Gibco). Explants were incubated overnight, then media was replaced with serum-free OptiMEM (Gibco), rested for 3 hours, and treated.

### Fetal membrane explant treatments

Human FM explants were treated with either no treatment (NT) as a media control or with 20 μg/ml of high molecular weight Poly(I:C) (Invivogen; San Diego, CA) for 24 hours. We previously reported that at this dose Poly (I:C) triggers an inflammatory cytokine/chemokine response [24]. For inhibitor studies, human FMs were pretreated for 1 hour with either with media, the TLR7 inhibitor, IRS661 (5μM; 5′-TGCTTGCAAGCTTGCAAGCA-3’; made by the Keck Core, Yale University) [17], the TLR8 inhibitor, CU-CPT9a (10μM; #inh-cc9a; Invivogen); or the inhibitor of sEV biogenesis/release, GW4869 (0.1μM; R&D Systems; Minneapolis, MN). The inhibitors, IRS661, CU-CPT9a, and GW4869 had no effect on FM viability as determined by measuring LDH release using the CyQUANT LDH Cytotoxicity Assay (Life Technologies). In other experiments, FM explants were transfected using siPORT NeoFX (Invitrogen, Waltham, MA) with either a scramble control, the miRVana inhibitor to miR-146a3p, or the miRVana inhibitor to miR-21a, all at 100nM (ThermoFisher Scientific, Waltham, MA) for 24 hours as previously described [17], prior to NT or Poly(I:C) treatments. After 24 hours, FM supernatants and explants were collected and stored at −80˚C for subsequent analyses.

### FM cytokine/chemokine measurements

Human FM explant supernatants were collected and centrifuged to clear the supernatants of debris. Supernatants were measured for known markers of inflammation/chorioamnionitis [17]: IL-6, IL-1β, and IL-8 using a standard sandwich ELISA protocol. All ELISA kits were obtained from R&D Systems, Minneapolis, MN and run according to manufacturer’s protocol. Absorbance was measured using an iMark microplate absorbance reader (Bio-Rad) and reported as pg/ml.

### Small extracellular vesicle isolation and validation

Small extracellular vesicles (sEVs) were isolated from FM supernatants using ExoQuick-TC (Systems Biosciences LLC, Palo Alto, CA) per the manufacturer’s instructions. Briefly, exoquick was added to the sample at a 1:5 ratio, incubated overnight at 4^o^C, centrifuged at 1500g for 30 minutes and supernatants discarded. The isolated sEV pellet was either: 1) resuspended in 50μl of phosphate buffered saline (PBS, Thermo Fisher Scientific, Waltham, MA) for subsequent nanoparticle tracking analysis (NTA) and quantification using a Zetaview (Particle Metrix, Ammersee, Germany) set to room temperature, a laser wavelength of 488nm, a sensitivity of 80, and a shutter speed of 100; 2) directly lysed for RNA extraction using the SeraMir Exosome RNA Purification Column Kit (Systems Biosciences) as previously described [23]; or 3) directly lysed into sample buffer for Western blot analysis.

### Western blot analysis

For Western blot analysis, samples in gel loading sample buffer were boiled for 5 mins, after which they were resolved under reducing conditions on 10% SDS-PAGE gels and then transferred onto PVDF membrane (PerkinElmer, Boston, MA). Membranes were blocked with 5% fat-free powdered milk (FFPM) in PBS/0.05% Tween-20 (PBS-T). Following washes with PBS-T, membranes were incubated overnight at 4°C with one primary antibodies in PBS-T/1% FFPM directed against either CD9 (#13174, Cell Signaling Technology; Danvers, MA), CD81 (#56039T, Cell Signaling Technology), CD63 (#52090, Cell Signaling Technology), or actin (#A2066, Sigma Aldrich). Following this incubation, membranes were washed as before and then incubated with the goat anti-rabbit IgG secondary antibody conjugated to peroxidase (Vector Labs; Burlingame, CA) in PBS-T/1% FFPM. Following washes with PBS-T and then with distilled water, the peroxidase-conjugated antibody was detected by enhanced chemiluminescence (PerkinElmer) and images were captured using an Amersham Imager 680 (General Electric, Boston, MA).

### RT-qPCR

RNA was isolated from FM tissue explants as previously described [17]. Tissue and sEV RNA concentrations were measured with a NanoDrop 2000 Microvolume Spectrophotometer (ThermoFisher Scientific; Waltham, MA). For reverse transcription of miR-21a, miR-29a, miR-146a-3p, and Let-7b, 30ng of sEV RNA or 1 μg of tissue RNA was added to the TaqMan MicroRNA Assay reverse transcription mix consisting of the target specific RT microRNA primer (Life Technologies; Carlsbad, CA), 100mM dNTPS, MultiScribe reverse transcriptase, reverse transcription buffer, RNAse inhibitor, and RNAse/endotoxin free molecular grade water, mixed gently, centrifuged, then incubated 30 minutes at 16^o^C, 30 minutes at 42^o^C, 5 minutes at 85^o^C as directed by the manufacturer. Each reverse transcription batch contained a sample without reverse transcriptase as a mock control for DNA contamination. For miR-146a-3p, a pre-amplification step was included prior to performing PCR using the Taqman PreAmp master mix kit (Life Technologies). To measure expression of miR-21a, miR-29a, miR-146a-3p, and Let-7b, qPCR was performed on cDNA with the TaqMan MicroRNA Assay (Life Technologies) according to manufacturer’s protocols. Briefly, 10μl of TaqMan 2X Universal PCR Master Mix (Life Technologies), 7.7μl of RNAse/endonuclease free water, 1μl of target specific 20X TaqMan MicroRNA Assay mix (Life Technologies), and 1.3μl of cDNA were mixed per sample/reaction and run in duplicate. No-template controls and mock cDNA samples were included for each target and experiment. Each plate was run for 10 minutes at 95^o^C, then 40 cycles of 95^o^C for 15 seconds and 60^o^C for 60 seconds. Small nuclear RNA U6 was a reference gene for normalization and data were analyzed with the 2^-ΔΔCT^ method and reported as fold change (FC) with 95% confidence intervals (CI).

### Statistics

For each human FM sample, explant treatments were performed in 3 technical replicates, pooled, and reported as one experiment. Experiments were performed in three or more biological/independent replicates and reported in the figure legends as “n=”. All analysis was performed in duplicate and averaged, and data are reported as mean ± SEM of independent experiments with statistical significance defined as *p* < 0.05. Statistical analysis was performed using Prism Software (Graphpad, La Jolla, CA). Normality was determined using the Shapiro-Wilks test. For normally distributed data, data were analyzed using either one-way analysis of variance (ANOVA) for multiple comparisons or a t-test. For data not normally distributed, data were analyzed using a non-parametric multiple comparison test or the Wilcoxon matched-pairs signed rank test. Outliers were determined by the ROUT method Q coefficient of 1%. When available, fetal sex was confirmed as an unsignificant variable as determined by multiple linear regression models. Additional statistical analyses are specified in table legends.

## Results

### Poly(I:C) does not increase FM tissue expression of TLR7/8-activating miRs

**Fig 1:**
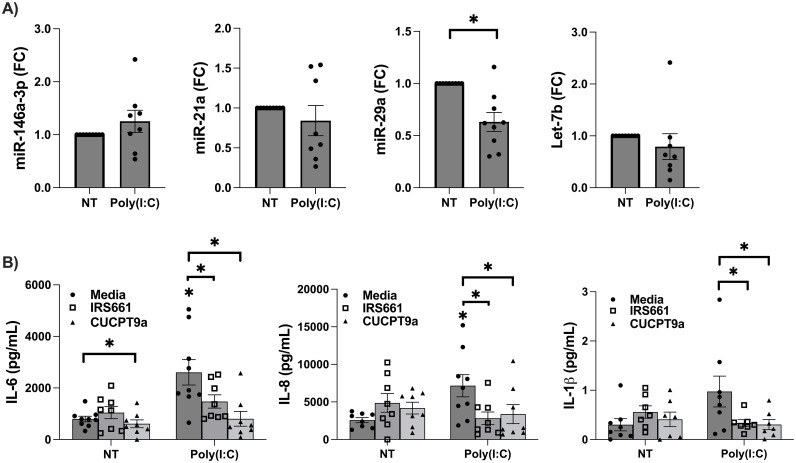
Poly(I:C) induces FM inflammation in a TLR7- and TLR8-dependent manner without elevating FM tissue expression of TLR7/8 activating microRNAs. **(A)** Human FM explants were either not treated (NT) as media controls or treated with Poly(I:C) for 24 hours and then tissues were collected for RNA extraction. The TLR7/8-activating miRs, miR-146a-3p, miR-21a, miR29a, and Let7b, were measured by RT-qPCR (n = 9). *p < 0.05. **(B)** FM explants were pretreated with media, the TLR7 inhibitor, IRS661, or TLR8 inhibitor, CU-CPT9a, for 1 hour. FMs were then untreated (NT) or treated with Poly(I:C) and after 24 hour supernatants were measured for pro-inflammatory IL-6, IL-8, and IL-1β (n = 8-9). *p < 0.05 relative to the NT/Media control unless otherwise indicated by brackets.

To investigate the role of TLR7/8-activating miRs in a viral model, FM explants were exposed to a viral dsRNA mimic, Poly(I:C), and the tissues measured for the TLR7/8-activating miRs, miR-146a-3p, miR21a, miR-29a and Let-7b [19–21]. Following Poly(I:C) stimulation, expression levels of miR-146a-3p, miR-21a, and Let7b were all unchanged compared to the NT controls, and miR-29a levels were significantly decreased by 38.6 ± 7.9% (CI: −0.6 – −0.2-fold; *p* < 0.05) (Fig 1A).

### Poly(I:C) induces FM inflammation in a TLR7- and TLR8-dependent manner

Human FM explant stimulation with Poly(I:C) (media-control) significantly increased the secretion of pro-inflammatory cytokine IL-6 by 3.4 ± 0.6-fold, the neutrophil recruiting chemokine IL-8 by 2.6 ± 0.4-fold, and the pro-inflammatory cytokine IL-1β by 6.9 ± 2.1-fold when compared to NT media control (Fig 1B). To investigate TLR7- and TLR8-dependent intermediate signaling previously reported in our bacterial models [13,17], FMs were pre-treated with either a TLR7 inhibitor, IRS661, or a TLR8 inhibitor, CU-CPT9a. In Poly(I:C) stimulated FMs, inhibition of TLR7 significantly reduced FM secretion of IL-6 by 33.8 ± 10.3%, IL-8 by 54.0 ± 11.1%, and IL-1β by 51.1 ± 13.9%, when compared to Poly(I:C) alone (Fig 1B). In the presence of the TLR8 inhibitor, CU-CPT9a, Poly(I:C) stimulated FM secretion of IL-6, IL-8 and IL-1β were significantly reduced by 66.1 ± 10.6%, 52.1 ± 11.3%, and 64.3 ± 12.0%respectively, when compared to Poly(I:C) alone (Fig 1B).

### Poly(I:C) stimulated FMs release sEVs expressing elevated miR-21a and miR-146a-3p

**Fig 2:**
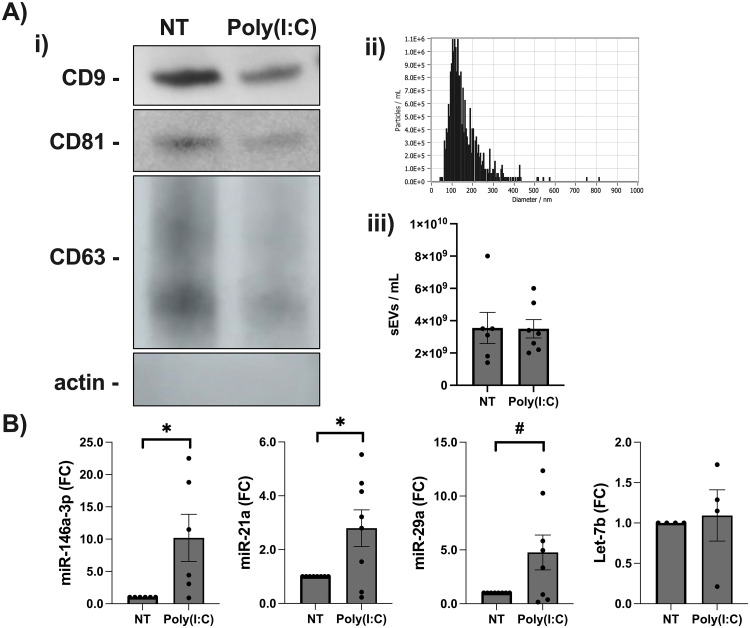
FMs treated with Poly(I:C) release sEVs containing elevated miR-146a-3p and miR-21a. **(A)** sEVs were isolated from the supernatants of FM explants treated for 24 hour with either NT or Poly(I:C). **i)** Image shows sEV expression of the surface markers CD9 (22-25 kDa), CD81 (22-25 kDa), and CD63 (40-60 kDa), and no actin expression, as determined by Western Blot. **ii)** Graph shows sEV size from a representative NTA run; and **iii)** Chart shows sEV concentrations as determined by NTA (n = 7). **(B)** RNA was isolated from sEVs and measured for the TLR7/8-activating miRs, miR-146a-3p, miR-21a, miR-29a and Let7b by RT-qPCR (n = 4-8; *p < 0.05; #*p* = 0.05).

With FM inflammation stimulated by Poly(I:C) being dependent on both TLR7 and TLR8, small extracellular vesicles (sEVs) were considered as an alternate method of TLR7/8-activating miR delivery. Thus, sEV release by FM explants and their miR cargo were investigated. The presence of sEVs in FM supernatants was confirmed by Western Blot for the sEV markers CD81, CD9, and CD63 (Fig 2A i). Furthermore, the possibility of cellular debris contamination of the sEV preparations was excluded by probing for actin, which was not detected (Fig 2A i). The presence of sEVs in FM supernatants was further validated using nanoparticle tracking analysis which confirmed sEV size (Fig 2A ii). As shown in Fig 2A (iii), FMs released similar amounts of sEVs under both NT and Poly(I:C) conditions. Treatment of FMs with Poly(I:C) significantly elevated sEV expression of miR-146a-3p and miR-21a by 10.2 ± 3.6-fold (CI: −0.2–18.6-fold) and by 2.8 ± 0.7-fold (CI: 0.2–3.4-fold), respectively (Fig 2B). sEVs from Poly(I:C) treated FMs expressed 4.8 ± 1.6-fold (CI: −0.1–7.6-fold) more miR-29a when compared to the NT control, however, this was not quite significant (*p* = 0.05, Fig 2B). Let7-b expression was unchanged in sEVs released from Poly(I:C) treated FMs when compared to no treatment (NT) controls (Fig 2B).

### FM IL-6 and IL-8 secretion in response to Poly(I:C) is dependent on sEVs

**Fig 3:**
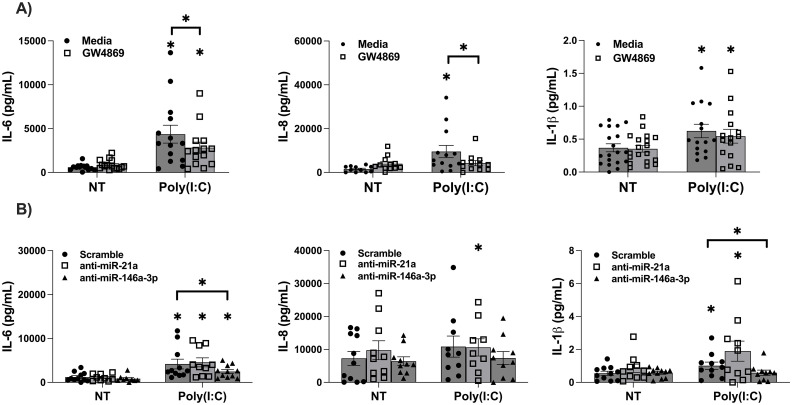
FM Poly(I:C)-induced pro-inflammatory factor release is partially dependent on sEVs and miR-146a-3p. **(A)** FM explants were pretreated with media or with the inhibitor of sEV biogenesis/release, GW4869, for one hour, and then treated with NT or Poly(I:C). After 24 hours, supernatants were measured for IL-6, IL-8 and IL-1β. (n = 14−19) *p < 0.05 compared to the NT media control unless otherwise specified. **(B)** FM explants were transfected with either a scramble control, an anti-miR-146a-3p inhibitor, or an anti-miR-21a inhibitor for 24 hours and then treated with NT or Poly(I:C) for another 24 hours after which supernatants were measured for IL-6, IL-8 and IL-1β (n = 11) *p < 0.05 compared to the NT control for each group unless otherwise specified by brackets.

The dependency of Poly(I:C) induced inflammation on sEVs was investigated using the inhibitor of sEV biogenesis/release, GW4869 [25]. The presence of GW4869 significantly reduced the ability of Poly(I:C) to induce FM secretion of IL-6 and IL-8 by 28.9 ± 6.3% and 40.7 ± 9.2%, respectively (Fig 3A). In contrast, Poly(I:C)-induced FM IL-1β secretion was unchanged by sEV inhibition (Fig 3A). As shown in S1 Fig, GW4869 significantly reduced sEV miR-21a expression.

### Poly(I:C)-induced FM secretion of IL-6 and IL-1β is dependent on miR-146a-3p

The role for TLR7/8-activating miRs was examined by transfecting FM tissues with specific miR inhibitors as previously reported [17]. The presence of the anti-miR-146a-3p inhibitor significantly reduced the ability of Poly(I:C) to trigger FM IL-6 secretion by 28.8 ± 9.9%, and IL-1β by 38.7 ± 9.8%, while IL-8 secretion was unchanged (Fig 3B). The anti-miR-21a inhibitor did not significantly alter FM secretion of IL-6, IL-8, or IL-1β in response to Poly(I:C) when compared to scramble controls (Fig 3B).

## Discussion

Bacterial infections are well established as contributors to chorioamnionitis and preterm birth, a major threat to the health of the mother and child. Viral infections are also known to be contributors to chorioamnionitis and preterm birth; however, studies on the link and mechanisms between viral infections and preterm birth are lacking. While most viral research associated with preterm birth and maternal/fetal outcomes has focused on TORCH pathogens, less is known about non-vertically transmitted viruses that can still result in inflammation and preterm birth, such as influenza and SARS-CoV-2 [4]. Previous studies using bacterial models of chorioamnionitis revealed that in response to PDG or LPS, miR-146a-3p is elevated in FM tissues and propagates inflammation in a TLR8-dependent manner both *in vitro* and *in vivo* [13,17]. In the current study, we investigated the role of this and other TLR7/8-activating miRs as intermediate regulators of FM inflammation in response to Poly(I:C), a viral dsRNA mimic.

Our group previously reported that human FMs secrete an inflammatory cytokine/chemokine response to the dsRNA mimic, Poly(I:C). However, the cytokine/chemokine profiles as measured using a multiplex protein assay did not include elevated IL-1β, IL-6, and IL-8, which our current study focused on [24]. We believe this discrepancy is due to the multiplex assay having lower sensitivity/specificity than individual ELISAs, which were used in this current study. In this current study we focused on upstream signals of Poly(I:C)-induced fetal membrane inflammation and sought to determine if TLR7/8-activating miRs played a role in this virally-mediated response. We first measured tissue expressed TLR7/8-activing miRs in FM explants. Surprisingly, and in contrast to our bacterial models [13,17], none of the four miRs screened were increased. In fact, miR-29a expression was significantly decreased following exposure to Poly(I:C). Nonetheless, using individual ELISAs, we found that Poly(I:C)-induced inflammatory IL-1β, IL-6, and IL-8 secretion was dependent on TLR7 and TLR8 activation. In particular, as we have previously reported [13,17], FM TLR8 had a role in this inflammatory response. Interestingly, in contrast to the bacterial model, TLR7 also played a role in mediating FM inflammation in response to Poly(I:C). Having established that both ssRNA sensors, TLR7 and TLR8, were playing a role in mediating downstream FM inflammation that was initiated by TLR3 activation in response to Poly(I:C), an alternative route of TLR7/8-activating miR delivery was investigated.

sEVs are important physiological communicators in health and disease; functioning to carry lipids, nucleic acids, and proteins from their cell of origin to neighboring cells or systemically to other organs [22]. In pregnancy, sEVs serve as critical mediators of maternal physiological and immune changes as the pregnancy progresses. While sEVs have important functions to facilitate fetal development and the maintenance of pregnancy, they are also contributors to pregnancy complications such as preeclampsia and preterm birth [26]. Animal studies have shown that sEVs are a method of maternal-fetal communication and can induce preterm birth, hypothesized to be through inflammatory and oxidative stress signaling [27–29]. In a mix of cellular and animal studies, the importance of sEVs in maternal-fetal inflammation and the robust ability for sEVs to influence preterm birth is clear [27,30–32]. Despite this, identification of sEV cargo consistently involved in maternal-fetal inflammation and their specific pathways have remained elusive, especially in viral models. With the lack of an elevation in locally tissue produced TLR7/8-activating miRs in FMs treated with Poly(I:C), we investigated sEVs as a source of these DAMPs. Small EVs (sEVs) were isolated from FM conditioned media and confirmed via Western Blot for the positive sEV membrane markers, CD9, CD81 and CD63, as well as by NTA. TLR7/8-activating miRs, miR-146a-3p and miR-21a, were found to be elevated in sEVs released from FMs exposed to Poly(I:C). Thus, we hypothesized that following viral TLR3 stimulation, FMs package elevated miR-146a-3p and miR-21a directly into sEVs to be released to target cells where they are unpackaged for miR activation of TLR7 and TLR8 for the propagation of inflammation. These FM-derived sEVs may provide an important mode of communication in viral TLR3-mediated chorioamnionitis and possibly in fetal inflammation since these sEVs can be released into the amniotic fluid. Indeed, TLR7/8-activating miRs are known to be packaged in sEVs and can exert stimulatory and inflammatory effects on target cells [19,20,23]. Furthermore, in a study measuring sEV miRs in preterm neonates, the group found that in neonatal bronchopulmonary dysplasia, sEV miR-21 was elevated with potential pro-inflammatory effects [33]. While FM inflammation was not studied alongside neonatal inflammation, the presence of miR-21 in fetal sEVs suggests a potential method of inflammatory communication between maternal and fetal tissues.

We predicted that in our current study, FM-derived sEVs were acting back in an autocrine manner to deliver TLR7/8-aciving miRs in order to propagate the inflammatory response to viral Poly(I:C). Thus, first to investigate the dependency of Poly(I:C)-induced FM inflammation on sEVs, we utilized the sEV biogenesis/release inhibitor, GW4869. GW4869 reduced the ability of Poly(I:C) to elevate FM secretion of both IL-6 and IL-8 in response to Poly(I:C). Interestingly, with sEV inhibition, Poly(I:C) induced IL-8 was reduced to baseline levels suggesting that IL-8 is mostly dependent on sEVs, while the IL-6 and potentially the IL-1β response may be only partially dependent upon this mechanism. Next, to investigate the dependency of Poly(I:C)-induced FM inflammation on the TLR7/8-activating miRs themselves, FM tissues were transfected with specific miR inhibitors. We found, somewhat surprisingly, that only miR-146a-3p appeared to regulate the FM IL-6 and IL-1β response to Poly(I:C). It is important to note that in these experiments, FM IL-8 secretion was not significantly increased with Poly(I:C) treatment in the scramble controls when compared to NT scramble controls, suggesting that the transfections may have altered or dampened the IL-8 response. While miR-146a-3p and sEVs have direct effects on FM inflammation, in our model, miR-21a may be produced in response to an inflammatory stimuli, but may have a role independent of the generation of inflammation. Indeed, studies found that sEV miR-21a had cardio and lung-protective roles, and it was hypothesized to downregulate expression of pro-apoptotic/necroptotic genes [34,35]. Alternatively, it is possible that sEV miR-21a does have a pro-inflammatory role on factors or tissues that were not measured in the current project. Several studies suggest that sEV miR-21a have roles in intestinal sepsis [36], macrophage polarization [37,38], and neuroinflammation [39], highlighting pro-inflammatory roles for sEV miR-21a. Further studies are needed to elucidate the role of FM-derived sEV miR-21a. It is also possible that the FM-derived sEVs under Poly(I:C) conditions contain other TLR7/8-activating DAMPs that we have yet to identify.

Limitations of this study include the variable nature human FM tissues and of Poly(I:C) as a TLR3 agonist. TLR3 recognizes dsRNA viruses; however, dsRNA viruses are limited in nature. More frequently, TLR3 senses dsRNA intermediates generated during the replication of ssRNA viruses [40]. This, in turn, may make TLR3 a robust secondary signaling mechanism, but a weak initial signal. Despite this, Poly(I:C) is a good viral mimic to test our hypothesis of miRs acting as DAMPs and secondary signals via TLR7 and TLR8 in FM inflammation.

With ongoing research efforts, the effects of viral infections on pregnancy outcomes are becoming clearer. Despite this, the mechanisms involved in the maternal viral response and their role in preterm birth is not well understood. Herein, we have demonstrated that TLR7/8-activating miR-146a-3p is an important pro-inflammatory DAMP mediating FM responses to a viral stimuli. We propose that upon recognition of a virus, FM miR-146a-3p is elevated and packaged into sEVs for delivery to neighboring cells where it perpetuates inflammation via TLR7 and TLR8 activation in and autocrine and paracrine manner within the tissue. Identification of this novel anti-viral signaling mechanism provides an opportunity for further exploration and a possible diagnostic or intervention step to mitigate inflammation induced preterm birth.

## Supporting information

S1 Fig FM explants were treated with no treatment (NT) or GW4869. After 24 hours, sEVs were isolated from supernatants, RNA was extracted, and miR-21a measured by RT-qPCR and shown as relative expression (RE) (n = 7) *p < 0.05.
(TIF)

S1 File Raw Western Blot Image Files. (PDF)

S2 File Raw Data. (XLSX)
