# Supplementary figures and images for: Viral dsRNA triggers human fetal membrane miR-146a-3p to be packaged into small extracellular vesicles which in turn drives inflammation through activation of Toll-like Receptor 7 and 8
Source: PLoS One. 2026 May 26;21(5):e0350139. doi: 10.1371/journal.pone.0350139 (PMC13210296; doi:10.1371/journal.pone.0350139)

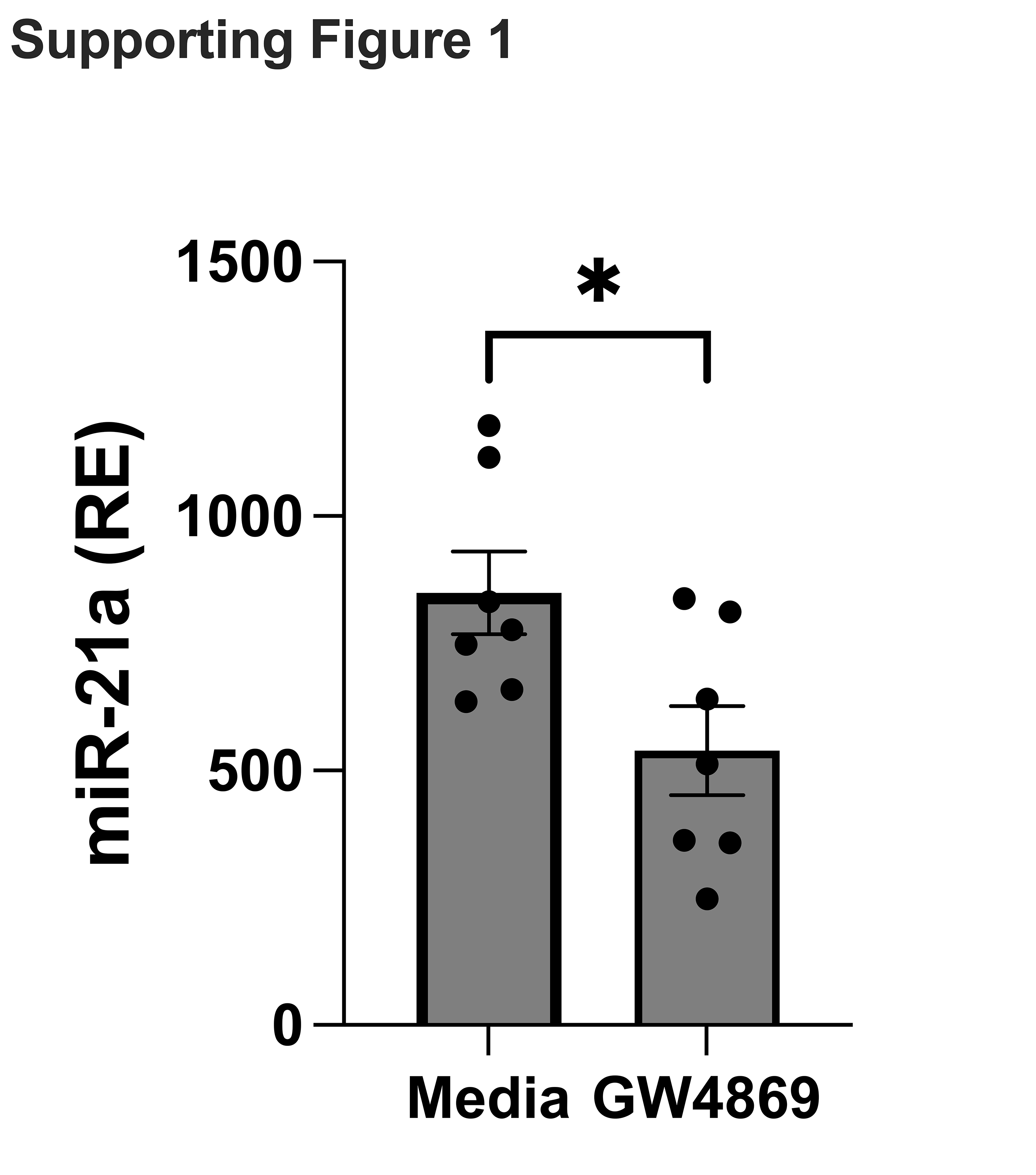

Supplement: S1 Fig — After 24 hours, sEVs were isolated from supernatants, RNA was extracted, and miR-21a measured by RT-qPCR and shown as relative expression (RE) (n = 7) *p < 0.05. (TIF) [file pone.0350139.s001.tif]
