# Supplementary material for: Viral dsRNA triggers human fetal membrane miR-146a-3p to be packaged into small extracellular vesicles which in turn drives inflammation through activation of Toll-like Receptor 7 and 8
Source: PLoS One. 2026 May 26;21(5):e0350139. doi: 10.1371/journal.pone.0350139 (PMC13210296; doi:10.1371/journal.pone.0350139)

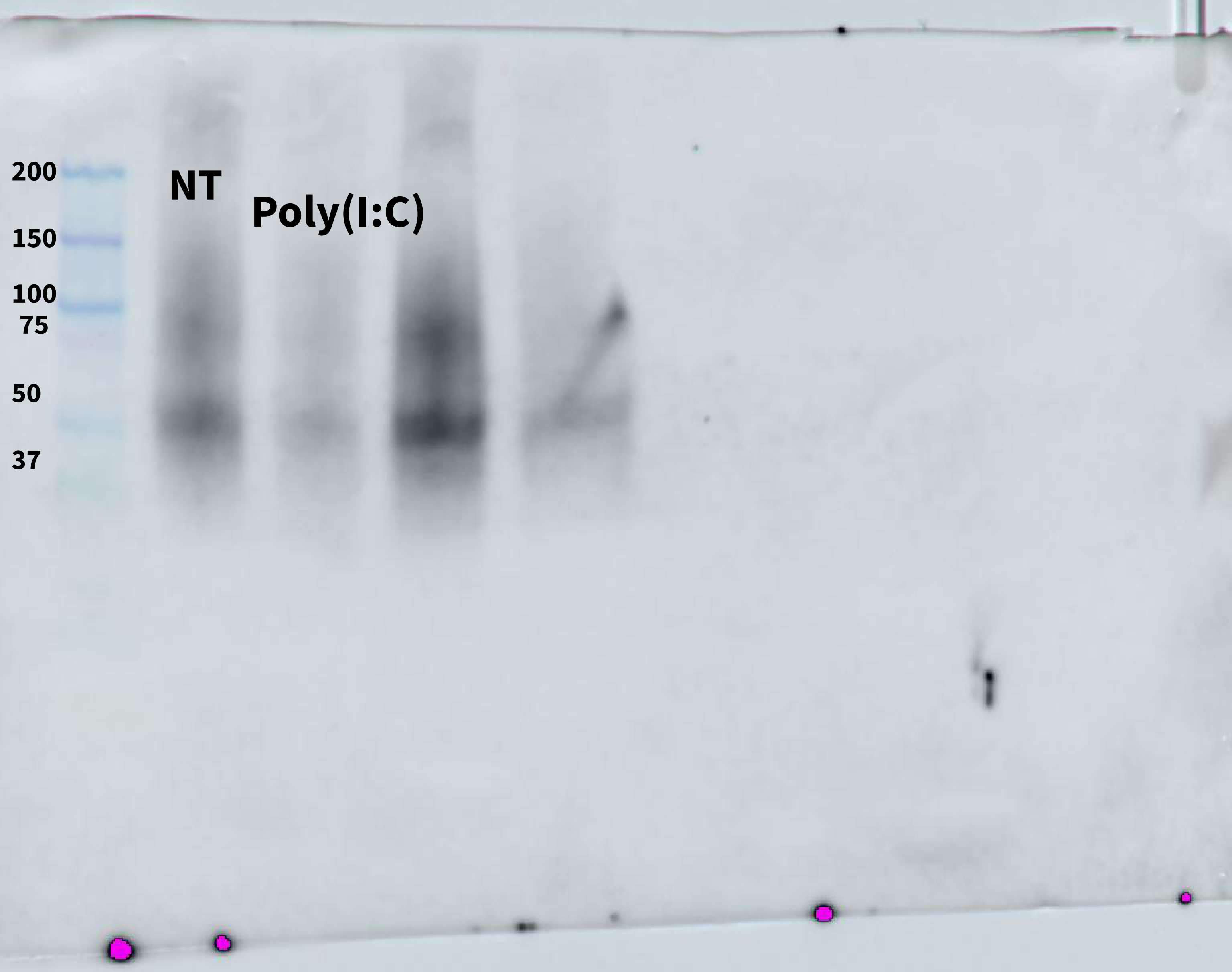

CD63 52090 1:1000 in 1% milk
3 min 30s exposure

CD81 56039T
1:1000 1% Non-fat milk
4 second exposure

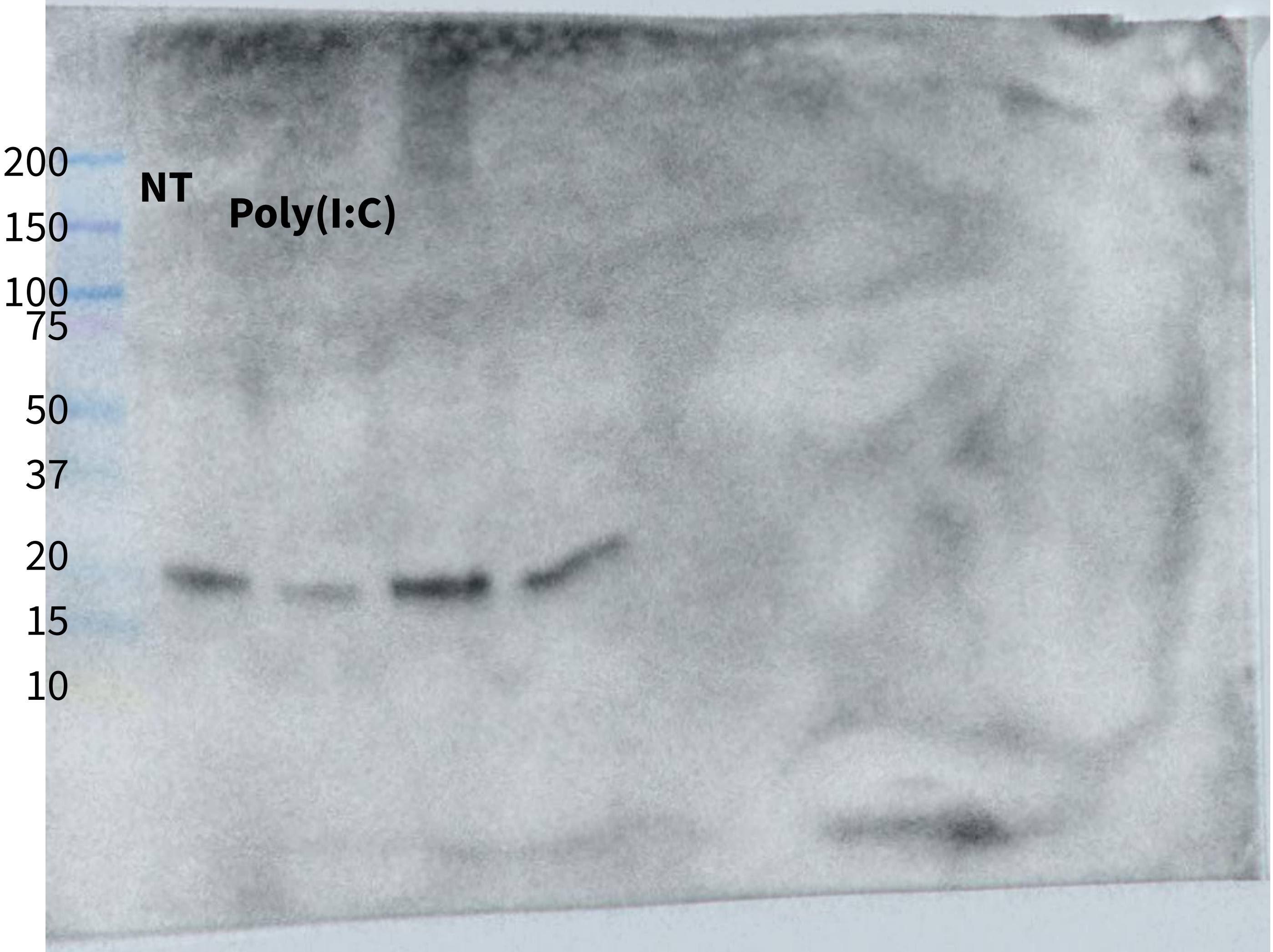

CD9 13174
1:1000 1% Non-fat milk
0.5 second exposure

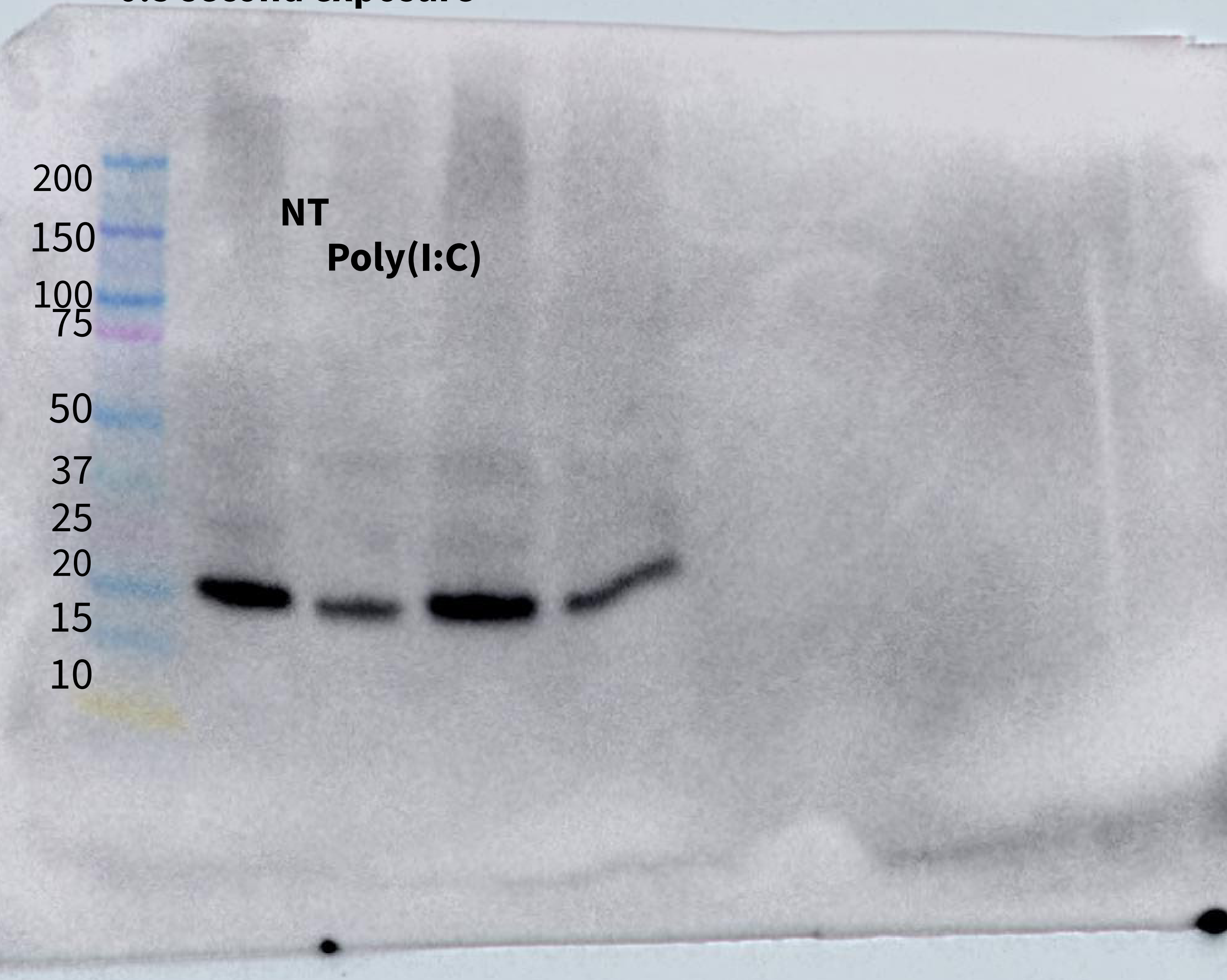

Actin A2066 1:10,000 1% milk
1 min exposure

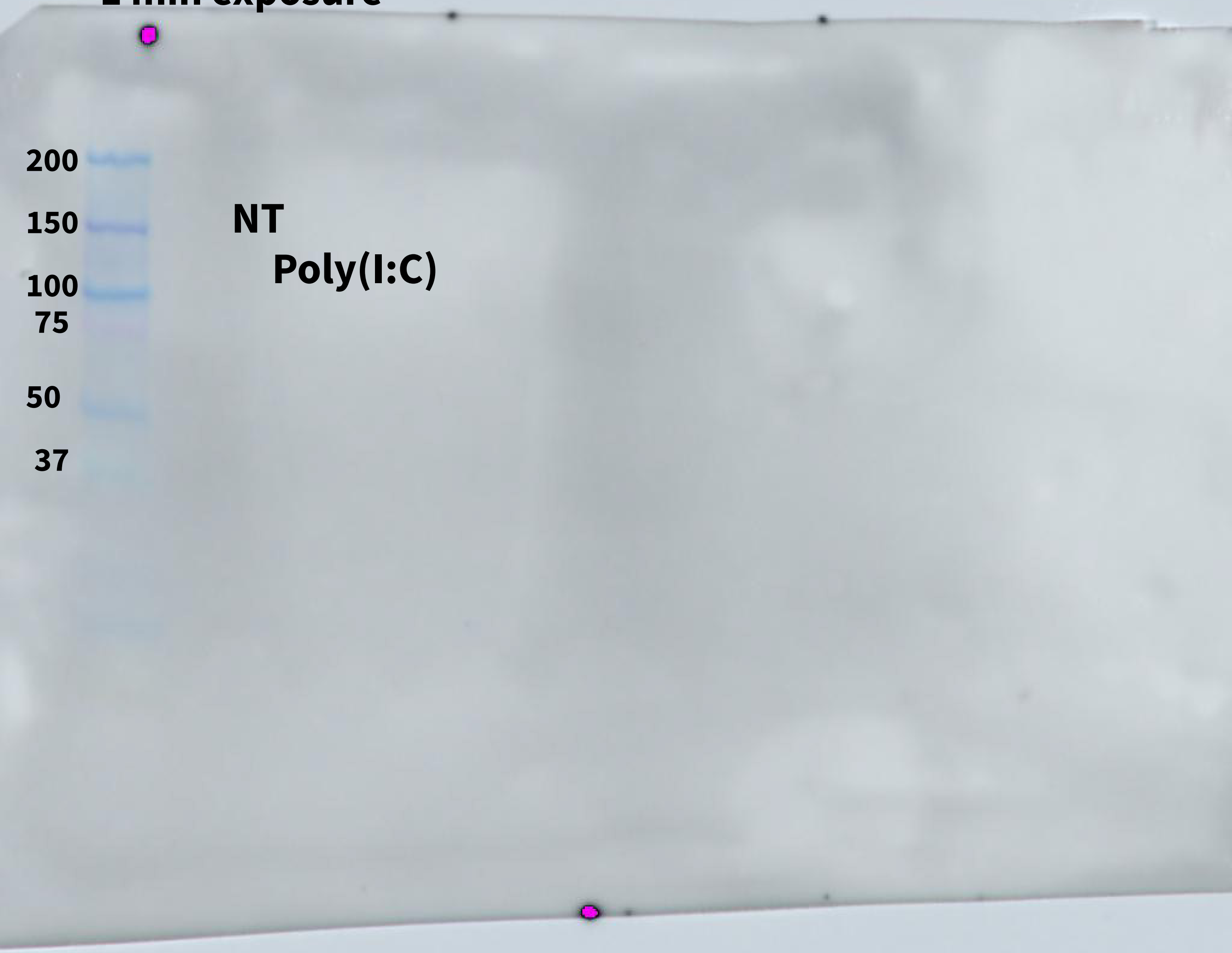

Supplement: S1 File — (PDF) [file pone.0350139.s002.pdf]
